# A Story of Abraham Lincoln

**Published:** 1882-05

**Authors:** 


### A STORY OF ABRAHAM LINCOLN.

IN the darkest hour of the late civil war, a great question arose in a thriving northern town, which for the purposes of this article shall be called Pepperton. This question was nothing less than this: Which of two local henchmen shall be postmaster? The contest waxed fierce. Deputation after deputation rushed to Washington—saw the Congressman, the Senator, the Postmaster-General, the President himself—besought, insisted, badgered. The subordinate henchmen thronged the corridors of the Capitol by day, and the bar rooms by night. The attention of honorable members was besought for articles in the Perkins County *Herald* on one side, and the Pepperton *Register* on the other. Monster petitions were forwarded by either side—petitions all the more monstrous because most people signed both.

Meantime, the civil war dragged on with increasing horrors. Rivers of blood had flowed, billions of treasure had been flung into the abyss, when a good old Pepperton judge—a steady Presbyterian deacon—visited Washington to see what light he could get on national affairs. In due time he stood before President Lincoln. The judge was shocked at the careworn face of the President, tried to comfort him and said : "Mr. Lincoln, I am sorry to see you not looking so well as when you passed through Pepperton. You must not let the rebellion wear upon you. The Lord is with us. He will not permit slavery and disunion to conquer. He has purposes with this republic which——" "Oh, judge," said Mr. Lincoln, "it isn't the rebellion that is killing me it isn't the rebellion ; it is your plagued Pepperton post-office ?"

This utterance of Mr. Lincoln—which is historical—goes, like so many quaint sayings of his, far into the marrow of of the evil. Columns of argument could not so well reveal the effect of the present system upon the executive ; and the fun becomes grim earnest when one recalls the fate of Harrison and Garfield.

